# Dominance of Sterilization and Alternative Choices of Contraception in India: An Appraisal of the Socioeconomic Impact

**DOI:** 10.1371/journal.pone.0086654

**Published:** 2014-01-28

**Authors:** Isabel Tiago de Oliveira, José G. Dias, Sabu S. Padmadas

**Affiliations:** 1 Instituto Universitário de Lisboa (ISCTE-IUL), CIES–IUL, Lisboa, Portugal; 2 Centre for Global Health, Population, Poverty and Policy and Division of Social Statistics and Demography, University of Southampton, Southampton, United Kingdom; London School of Economics, United Kingdom

## Abstract

**Background:**

The recent decline in fertility in India has been unprecedented especially in southern India, where fertility is almost exclusively controlled by means of permanent contraceptive methods, mainly female sterilization, which constitutes about two-thirds of overall contraceptive use. Many Indian women undergo sterilization at relatively young ages as a consequence of early marriage and childbearing in short birth intervals. This research aims to investigate the socioeconomic factors determining the choices for alternative contraceptive choices against the dominant preference for sterilization among married women in India.

**Methods:**

Data for this study are drawn from the 2005–06 National Family Health Surveys focusing on a sample of married women who reported having used a method of contraception in the five years preceding the survey. A multilevel multinomial logit regression is used to estimate the impact of socioeconomic factors on contraceptive choices, differentiating temporary modern or traditional methods versus sterilization.

**Findings:**

Religious affiliation, women's education and occupation had overarching influence on method choices amongst recent users. Muslim women were at higher odds of choosing a traditional or modern temporary method than sterilization. Higher level of women's education increased the odds of modern temporary method choices but the education effect on traditional method choices was only marginally significant. Recent users belonging to wealthier households had higher odds of choosing modern methods over sterilization. Exposure to family planning messages through radio had a positive effect on modern and traditional method choices. Community variations in method choices were highly significant.

**Conclusion:**

The persistent dominance of sterilization in the Indian family planning programme is largely determined by socioeconomic conditions. Reproductive health programmes should address the socioeconomic barriers and consider multiple cost-effective strategies such as mass media to promote awareness of modern temporary methods.

## Introduction

Historically, the family planning program in India had focused heavily on the promotion of permanent methods in response to the need for controlling rapid population growth and widespread poverty [1]–[4]. The program initially emphasized on a number of modern methods [5] but later shifted its focus towards male sterilization [6]. The undue emphasis on sterilization targets, unscrupulous incentive-based administration, poor service standards and the coercive nature of the program created public haul and eventually led to the fall of the ruling government in 1977 [6]–[8]. Since the late 1970s, the program shifted its focus towards female sterilization which thereafter continued to dominate the Indian family planning program. Health workers vigorously promoted female sterilization to fulfil their targets and often misled women about other method choices [8]. In fact, the dominance of female sterilization undermined the promotion and use of other spacing methods and sterilization was often the only method of contraception in women's reproductive lives [9]. Added to this, are the stagnant rates of early marriage and childbearing and stopping behaviour facilitated by sterilization at relatively young ages [10]. The sterilization at very young ages is associated with sterilization regret, particularly for the women without male offspring and in the case of child loss [11].

An informed choices model of service delivery was introduced in 1998, emphasizing individual reproductive and family planning needs and rights, and offering quality services without any form of coercion or discrimination. Although the impact of informed choices cannot be directly measured, evidence shows changes in method mix among recent users [12]. Nevertheless, the extent to which they vary across different social groups is not well understood. Recent evidence shows inequalities in the provision of family planning and reproductive health information, particularly women from poorer communities are disadvantaged in terms of better care and information [13].

Poor and marginalized women typically have high unmet needs for modern contraception especially spacing methods, and as a result, they become vulnerable to experiencing poor reproductive outcomes including high rates of unintended and unwanted pregnancies [14]. Ethnic status is yet another source of discrimination for poor women from scheduled tribes, scheduled castes and other backward communities [15]. Poor women in Indian states generally have poor access to education and health care. These women usually have little autonomy within and outside the household and have little control over their own reproductive and contraceptive choices [16]–[17]. This is particularly the case among newly married women with poor education, who lack security and often surrender to the ideals, norms and expectations of their husbands and in-laws. For example, son preference is deeply rooted in the Indian culture and usually dictated by husbands and in-laws especially in joint households, independent of economic status [18]–[20].

We hypothesise that women from poor and marginal communities have little choices for modern temporary methods other than sterilization. This paper investigates the factors determining alternative contraceptive choices to the dominant preference for sterilization among married women in India, with a focus on new users. More explicitly, the analysis aims at examining the impact of cultural and socioeconomic heterogeneity on contraceptive choices and the associated factors.

The structure of this paper is as follows. Data and methods used in the testing of our hypotheses are discussed in the next section. Following this, the results are discussed. Then the paper concludes with key findings, limitations of the research, and suggested directions for further research.

## Data and Methods

### Data

Data for this study were drawn from the third round of the National Family Health Survey (NFHS-3) conducted during 2005–06 in 29 states across 6 major geographic regions [12], retrieved from MEASURE DHS public repository (site: http://www.measuredhs.com/). The data set was provided for research use in anonymized version and hence no formal ethical approval was necessary.

NFHS-3 collected data on family planning, reproductive and maternal health, child health including anthropometric indicators, HIV related knowledge and relevant demographic and social data. The survey achieved a response rate of 94.5% and yielded high quality data including a nationally representative sample of 124385 women aged 15–49 and 74369 men aged 15–54. A two-stage sample design was adopted in rural areas with Primary Sampling Units (PSUs or villages) selected by means of probability proportional to population size and then a systematic selection of households within each PSU. The design for urban areas included a three-stage procedure to capture households within large wards based on census enumeration blocks. Further details of sampling design are published elsewhere [12].

### Analytical Sample

Since the aim of this analysis is to disentangle the factors behind the adoption of alternative contraceptive methods in a context characterized by the strong dominance of female sterilization, we included only women who reported using contraception at the time of survey. The exclusion of the non-users is a necessary condition to identify the impact on the choice between different methods, isolating the models from the effects of use versus non-use of contraception. We did examine for potential difference in characteristics between users and non-users (not shown separately). The results confirmed the socioeconomic gap in the uptake of contraception, especially modern methods [17],[21]–[23].

The analysis considered a sub-sample of married women who reported initiating method use during the five years prior to the date of interview. The focus on new users would enable the analysis to capture recent trends in method choices and associate contraceptive use with important current status variables such as household wealth, mass-media exposure or self-reported autonomy in healthcare decision making. About 42% of married women aged 15–49 reported not using a method in the five years preceding the survey (*n* = 32735). This yielded a sample of 19675 new users during the observation window.

### Outcome Variable

The dependent variable is the current contraceptive method classified into three categories: sterilization, other modern methods, and traditional ones. Female sterilization was considered the reference category in order to make meaningful comparison of the factors determining alternative method choices. The other two categories in the analysis were traditional methods and modern spacing/temporary methods. Traditional methods included rhythm, withdrawal and folk methods whereas modern temporary methods included the pill, IUD, injectables, condom/nirodh or other modern method excluding male or female sterilization. The number of male sterilization cases in the NFHS were very few (<1%) for the observation period and hence excluded from the analysis.

### Explanatory Variables

The analysis included a range of selected cultural and socioeconomic characteristics of the respondent (religion, caste status, education, occupation and wealth), exposure to family planning in the media (radio, television and newspapers), household type (nuclear or joint), a proxy variable measuring women's autonomy based on her decisions for her own healthcare), demographic characteristics (age, number and sex composition of children), and place and region of residence. These variables were selected based on a review of existing literature more broadly within the Indian context [9],[17],[21],[24]–[28]. Additionally, we considered husband characteristics as education, occupation and age (results not shown separately). The variables were initially screened for problems of multicollinearity before including those in the regression models.

### Methods

This study focused exclusively on recent users of modern temporary, permanent and traditional methods. The aim of the analysis was to identify the factors that reinforce the chances to adopt modern temporary family planning methods instead of the dominant method in comparison with other traditional contraception. The analysis considered a multilevel multinomial logit model with type of method used as the dependent variable, taking into account the hierarchical nature of the dataset with women (level 1) nested within communities (level 2). The multilevel multinomial model can be expressed as:
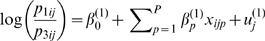
(1)

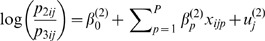
(2)

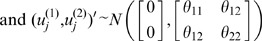
(3)where *p_1ij_*_,_ *p_2ij_* and *p_3ij_* represent the probability of using a traditional method, modern (temporary) method and permanent (sterilization) respectively. Sterilization (*p_3ij_*) is the reference category. contains values for independent variables and and are intercept and slope regression parameters, respectively. and refer to random error component in odds in equation (1) and (2), respectively. This specification of the model circumvents the axiom of Independence from Irrelevant Alternatives (IIA) needed to derive multinomial logit regression [29]–[30]. This essentially implies in the multinomial model the likelihood of women who are not using other modern temporary methods would use a traditional or a permanent (sterilization) method in the expected proportions. Adding this bivariate normal random effect would suggest that the odds are no longer independent on other possible outcomes, i.e., alternatives are no longer irrelevant as we take distinct random effects for each ‘odds’. The covariance measures the dependence between the odds within a multilevel framework. This model extends the binary case in to the multinomial one [31].

## Results

### Sample Characteristics

Table 1 shows the characteristics of new users who initiated a method during the five years preceding the survey. Permanent methods represented 43% of overall method use, modern temporary methods 34% and traditional methods constituted to about 23% of overall method use.

**Table 1:** Contraceptive user characteristics and method mix (*n* = 19675), India, 2005–06.

			Method mix conditional on use (%)			
	Number of users	% of users	Traditional	Modern	Sterilization	Total
**Women's caste**						
Scheduled caste	3209	19.4	24.4	27.1	48.5	100.0
Scheduled tribe	2054	6.8	20.4	22.6	57.0	100.0
Other backward caste	5973	38.6	20.2	28.3	51.5	100.0
General	7438	35.2	22.6	46.1	31.3	100.0
**Women's religion**						
Hindu	14583	80.4	21.7	31.2	47.1	100.0
Muslim	2724	14.3	28.6	47.7	23.7	100.0
Other	2368	5.3	19.5	41.6	38.9	100.0
**Women's education**						
None	5782	37.3	25.0	21.8	53.2	100.0
Primary	2617	14.3	21.2	29.5	49.3	100.0
Secondary	8602	38.8	21.6	40.6	37.8	100.0
Higher and above	2673	9.7	19.1	61.9	19.0	100.0
**Women's occupation**						
Professional skilled	1032	3.6	18.5	54.0	27.5	100.0
Services skilled manual	2781	12.9	19.8	30.8	49.4	100.0
Agricultural	3322	21.7	21.2	15.6	63.2	100.0
Not working	12520	61.8	23.8	40.1	36.1	100.0
**Household wealth**						
Poorest	1986	16.1	29.6	19.2	51.2	100.0
Poorer	2644	18.4	26.9	21.5	51.6	100.0
Middle	3531	18.4	22.7	26.2	51.1	100.0
Richer	4842	21.6	19.0	37.9	43.1	100.0
Richest	6672	25.5	17.8	55.1	27.1	100.0
**Heard FP on radio**						
Yes	7404	35.4	24.1	39.5	36.4	100.0
No	12271	64.6	21.7	31.1	47.2	100.0
**Heard FP on TV**						
Yes	12190	54.4	19.2	43.3	37.5	100.0
No	7485	45.6	26.6	23.1	50.3	100.0
**Heard FP on newspaper**						
Yes	6299	25.5	19.0	50.5	30.5	100.0
No	13376	74.5	23.8	28.5	47.7	100.0
**Household type**						
Nuclear	9976	49.9	22.3	29.8	47.9	100.0
Non-nuclear	9699	50.1	22.8	38.4	38.8	100.0
**Women's healthcare decision**						
Herself	5309	26.3	20.9	35.4	43.7	100.0
Spouse	5310	30.6	22.7	31.1	46.2	100.0
Jointly	958	6.0	25.6	36.1	38.3	100.0
Other	8098	37.0	23.1	35.3	41.7	100.0
**Women's age (in years)**						
15–25	4527	26.0	26.7	40.8	32.5	100.0
25–34	11878	58.9	18.6	32.0	49.4	100.0
35–49	3270	15.1	30.8	30.6	38.6	100.0
**Number of living children**						
0	565	2.9	53.8	45.4	0.8	100.0
1	3843	16.8	35.0	60.5	4.5	100.0
2	7192	35.3	16.3	33.5	50.2	100.0
3	3956	20.7	16.1	23.8	60.2	100.0
4+	4119	24.4	24.8	24.2	51.0	100.0
**Number of living sons**						
0	3825	17.5	35.1	47.5	17.4	100.0
1	8563	41.6	21.1	39.1	39.8	100.0
2	5265	28.7	15.8	22.3	61.9	100.0
3	1415	8.4	23.7	24.4	51.9	100.0
4+	607	3.8	29.1	28.8	42.1	100.0
**Region**						
North	3771	13.4	12.5	49.3	38.2	100.0
East	2958	22.9	30.9	36.3	32.8	100.0
Northeast	3747	5.2	50.4	37.9	11.7	100.0
West	2440	15.3	12.4	39.5	48.1	100.0
Central	3886	25.7	31.3	32.0	36.7	100.0
South	2873	17.5	7.1	16.9	76.0	100.0
**Place of residence**						
Urban	9604	35.6	17.5	47.4	35.1	100.0
Rural	10071	64.4	25.4	26.7	47.9	100.0
**All**	**19675**	**100.0**	**22.6**	**34.1**	**43.3**	**100.0**

Notes: percentages adjusted for sample weights; the number of users is based on unweighted data; Data source: Indian National Family Health Survey, 2005–06.

The cultural and social characteristics of users reveal key relations with the method choices. For instance, Hindu women who constituted the majority tend to choose permanent methods, whereas their Muslim counterparts rely on modern temporary or traditional methods. Social class differentiation in method choices is also apparent. There is a clear poverty gradient with regard to method choices. Women from marginal and backwards communities have less access to modern temporary methods. In particular, women belonging to scheduled tribes rely mostly on sterilization when compared to the general category. There is a positive association between women's education and modern temporary method choices. Highly educated women are about thrice as likely as those with no formal schooling to use modern temporary methods. A reversal trend is observed in the case of sterilization use. Sterilization use is common among women engaged in agricultural activities. In contrast, those working in professional skilled jobs tend to use modern temporary methods. Poor and middle class women increasingly rely on permanent methods whereas their richest counterparts tend to use modern temporary methods. The use of traditional method is common among poor women but less frequent amongst their richest counterparts.

Those who have accessed family planning information through media are considerably more likely to have chosen modern temporary methods whereas their counterparts are inclined to choose sterilization.

Sterilization choice is common among women belonging to nuclear households, whereas those in the non-nuclear households are equally likely to choose modern temporary methods and sterilization. The autonomy indicator which captures the decision making on healthcare shows that if a woman makes healthcare decision jointly with her spouse then she is inclined to choose sterilization. However, there is little difference in the choices when she makes the decision on her own.

Regarding the age of woman, clearly those in the middle ages 25–39 are likely to choose sterilization. Although their younger counterparts (15–25) are inclined to choose modern temporary methods, they are likely to have had not completed childbearing, except in a few south Indian states where childbearing is early and compressed in short intervals. There is a positive relationship between sterilization choice and number of living children, particularly living sons. Where couples do not have sons in the family, they are likely to have chosen modern temporary or traditional methods. However, about one-fifth of those without any sons choose sterilization.

Spatial characteristics show clear differences in method choices. Rural women who represent the majority are more inclined to choose sterilization whereas their urban counterparts favour modern temporary methods. As expected, the southern region dominates sterilization choices whereas modern temporary method choices are popular in the northern and western regions. About one half of the women in the northeast region choose traditional methods which in contrast is less than one tenth in the southern region.

**Figure 1:**
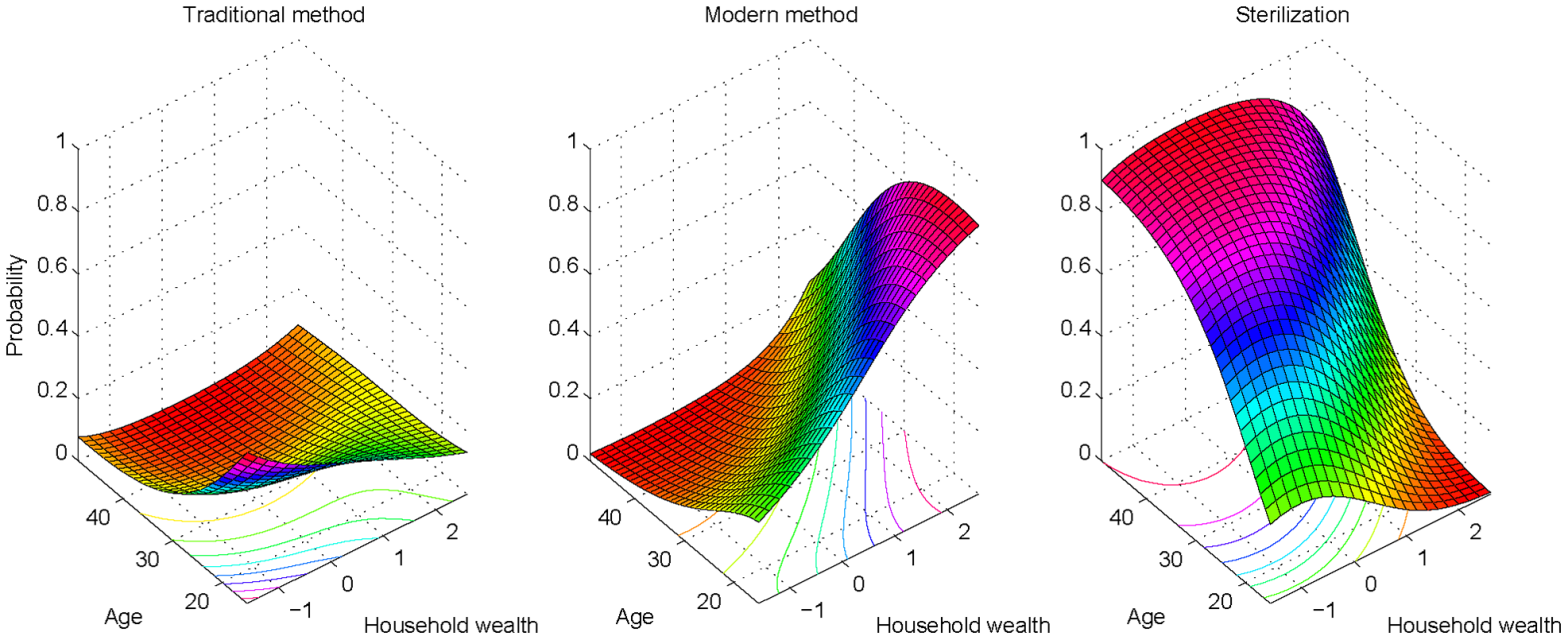
Probability of method use by household wealth and age, India, 2005–06.

We estimated predicted probabilities of method use adjusting only for wealth and age. The probability of a traditional method use is high among younger women belonging to poorer households whereas for a modern temporary method the probability is significantly high among younger women from wealthier households (Figure 1). In the case of sterilization, the probability is higher among older women belonging to the poorer households than those from wealthier households.

### Regression Analysis

The effect of selected variables associated with contraceptive choices is examined by a random intercept model with individual and community (Primary Sampling Unit) levels. Table 2 reports the adjusted odds ratios based on the multilevel multinomial logit regression. The reference category of the dependent variable method choices is sterilization, which is compared against traditional and modern methods. There are clear socioeconomic differences in method choices among Indian women.

**Table 2:** Odds ratios showing the effect of social variables on contraceptive choice, adjusting for relevant characteristics and random effects (*n* = 19675).

	Traditional vs. sterilization	Modern vs. sterilization
**Women's caste**		
General (ref)	1.000	1.000
Scheduled caste	0.965	0.990
Scheduled tribe	0.471***	0.760*
Other backward caste	0.872	0.943
**Women's religion**		
Hindu (ref)	1.000	1.000
Muslim	3.633***	5.207***
Other	0.976	1.190
**Women's education**		
None (ref)	1.000	1.000
Primary	0.868	1.130
Secondary	1.106	1.465***
Higher	1.456*	2.358***
**Women's occupation**		
Not working (ref)	1.000	1.000
Professional, skilled	0.772	0.709**
Service, skilled-manual	0.856	0.783**
Agricultural	0.846*	0.612***
**Household wealth**		
Poorest (ref)	1.000	1.000
Poorer	0.803*	0.908
Middle	0.713**	0.934
Richer	0.818	1.97
Richest	0.866	1.487**
**Respondent heard FP on radio**		
No (ref)	1.000	1.000
Yes	1.275***	1.177**
**Respondent heard FP on TV**		
No (ref)	1.000	1.000
Yes	0.823**	1.135*
**Respondent heard FP on newspaper**		
No (ref)	1.000	1.000
Yes	1.119	1.108
**Household type**		
Nuclear (ref)	1.000	1.000
Non-nuclear	1.054	0.989
**Women's health care decision**		
Both husband and wife (ref)	1.000	1.000
Wife	0.815**	0.880*
Husband	1.003	0.967
Other	0.930	0.868
**Women's age**		
35+ (ref)	1.000	1.000
15–25	0.679**	1.111
25–34	0.601***	0.812*
**No. of living children**		
0 (ref)	1.000	1.000
1	0.237*	0.399
2	0.008***	0.017***
3	0.005***	0.010***
4+	0.005***	0.010***
**No. of living sons**		
0 (ref)	1.000	1.000
1	0.463***	0.535***
2	0.239***	0.281***
3	0.295***	0.415***
4+	0.284***	0.450***
**Region**		
South (ref)	1.000	1.000
North	13.105***	22.421***
East	25.028***	14.154***
Northeast	111.052***	57.397***
West	5.155***	5.641***
Central	28.503***	17.814***
**Place of residence**		
Rural (ref)	1.000	1.000
Urban	0.803**	1.116

Note: *p<0.05; ** *p<*0.01; *** *p<*0.001; PSU level covariance was significant at 1%; Data source: Indian National Family Health Survey, 2005–06.

The random effects are significant at the community level suggesting considerable heterogeneity between communities in method choices among women in India. The estimate of the correlation between the random effects (standardized ) is 0.692 (*p*<0.001). As the IIA assumption is violated, the alternatives are relevant and traditional multinomial regression model cannot be applied. Our model extension, defining random effects in terms of odds, relaxes this assumption taking into account community effects.

The present analysis reveals considerable socioeconomic differences in contraceptive choices among married women who reported current using a contraceptive method.

As expected, the choice for contraceptive alternatives to sterilization depends strongly on the family size and region. Indeed, as sterilization is a definitive method, the offspring number and sex composition are among the most noticeable factors to explain the choice for alternative family planning methods [26]. The region of residence is a major factor as family planning policies have been implemented at the state level with distinct emphasis on sterilization [12]. After controlling for these elements including residence and other demographic variables, the influence of cultural and socioeconomic factors remain significant.

Regarding cultural and socioeconomic impacts on the choice of contraceptive methods, results confirm the expected associations. The longstanding heterogeneity of population of India associated with religion and the caste system is still relevant in contraceptive choices. It is clear that the major differences depend on religion, but not on the caste and tribe belonging. Muslim women tend to prefer traditional or modern temporary methods rather than sterilization, strongly associated with the comparatively low prevalence of sterilization, and high utilization of pill and condom [12]. On the other hand, women with other religious affiliation are not significantly different when compared with Hindu women. In contrast, sterilization odds increase in comparison with any other family planning methods for women belonging to the schedule tribes.

Women with secondary or higher level education are likely to prefer temporary methods over sterilization. The effects are highly significant for those who prefer to use modern reversible over permanent methods. Working women generally prefer sterilization instead of modern temporary methods and the same is true when the women consider themselves as responsible for their healthcare decisions. The wealthiest households are likely to prefer modern temporary methods over sterilization than their poorest counterparts. The trends are not linear as expected. As for the modern socioeconomic and cultural factors the foregoing analyses confirm our hypothesis that education and mass media exposure to family planning messages increase the chances to adopt modern temporary methods. Exposure to mass media messages on radio or television about family planning increases the chances to adopt modern temporary methods instead of sterilization.

There is no significant difference in method choice between type of household and method choice. Regarding healthcare decision making, when wives make decision on their own then they are likely to choose sterilization over other reversible methods when compared to those who make joint decisions with husbands. Younger women and those with one or more living sons are significantly less likely to use reversible methods. Women residing in southern region were significantly highly likely to choose sterilization over reversible methods when compared to their counterparts. The residence effect is marginally significant only for the traditional method category suggesting that urban women are less likely to prefer sterilization over traditional methods. However, they are likely to choose a modern reversible method than sterilization: the difference, however, is not statistically significant.

## Discussion

Although the use of modern temporary method has increased recently, the results from this study clearly demonstrate evidence of continuing sterilization dominance in the Indian family planning program [4]. The foregoing analyses confirm our hypothesis that women from poor and marginal communities continue to have less opportunities for modern method choices other than sterilization.

Socioeconomic dimensions have overarching influence on method choices, and the effects are not linear as expected. The primary variables – caste, religion, education, occupation and household wealth – show significant association with method choices and the patterns are fairly consistent across different socioeconomic groups. Sterilization choice is common among women from poor households; socially disadvantaged ethnic groups especially those belonging to schedule tribe communities, working women and those with little or no education. Those working in professional and skilled non-manual sector come mostly from the middle social class and are inclined to start childbearing relatively later than their counterparts, have small family size in a short period of time, and then limit fertility by choosing sterilization [10]. The positive effect of education on modern temporary method choices is widely acknowledged [16]–[17]. Educated women generally have better access and sufficient knowledge about the efficacy of modern temporary methods in preventing conceptions. Higher education also increases the odds to adopt a traditional method instead of sterilization, consistent with evidence from previous research [6].

The results presented in this study date to user experiences post-1998 when the government introduced a target-free approach to family planning services [4]. Clearly, the persistent dominance of sterilization use amongst the poorer strata of the Indian society highlights the weakness of the national family planning programme in promoting wider method choices. The exceptionally high use of sterilization in the southern region could be correlated with high demand for limiting fertility and better network of family planning services. In contrast, the physical access to reproductive health services is generally poor in larger high fertility states located in northern and eastern regions [13]. On the other hand, there are misconceptions about reversible methods and experience of side effects which deter women from using short-term reversible methods [32]–[33].

The main source for family planning in India is the public sector which is usually constrained in terms of manpower, training infrastructure, commodities and supplies [33]. Financial and related opportunity costs might exclude poor and uneducated women from accessing modern contraceptive methods. Modern contraceptives are available free of cost in public sectors including sterilization services. The NFHS-3 data show that about 90% of women who access sterilization services in public medical sectors availed the method without any financial payment [12]. The corresponding figures for IUD, pill and condom are 68%, 76% and 70% respectively. However, family planning services offered through public sectors primarily focus on promoting permanent methods and often lack quality of care in service delivery [32]. There is also evidence to suggest that even the poor in rural areas across Indian states are increasingly relying on private sectors for health care [34].

The present findings highlight the positive influence of female autonomy on sterilization choices, consistent with the observation that there is a constant cultural diffusion of sterilization knowledge and practice in Indian families across generations [28]. On the other hand, exposure to family planning messages through radio and television reveal a significant positive impact on contraceptive preference for temporary methods. The findings suggest that family planning messages promoted through mass media could be strengthened in socially disadvantaged communities to inform women and couples of the effectiveness of short-term reversible methods and its advantages in terms of spacing between births and associated protective effects for maternal and child health and survival.

A particular strength of the analysis is the data on recent users which enabled us to understand the current trends in contraceptive uptake among married women and their association with relevant socioeconomic indicators. The significant random effects at the community level suggest considerable variation in contraceptive demand and provision between communities. The present analysis could not disentangle whether there is any provider bias and social exclusion in family planning service delivery in India. Also, the analysis could not measure any direct effect of family planning messages promoted through mass media due to lack of information in the NFHS. Further qualitative evidence is needed to ascertain whether poor and socially deprived women are not fully informed of modern temporary method options other than sterilization. Further analysis of non-users is beyond the scope of this paper since the primary aim was to determine the skewness in method choices among users.
