# Correction: Dominance of Sterilization and Alternative Choices of Contraception in India: An Appraisal of the Socioeconomic Impact

**DOI:** 10.1371/journal.pone.0100050

**Published:** 2014-06-06

**Authors:** The PLOS ONE Staff

There is an error in the affiliation for the second author. The correct affiliation for José G. Dias is #3 Instituto Universitário de Lisboa (ISCTE-IUL), BRU Business Research Unit, Lisboa, Portugal.
